# Radiomic analysis of enhanced CMR cine images predicts left ventricular remodeling after TAVR in patients with symptomatic severe aortic stenosis

**DOI:** 10.3389/fcvm.2022.1096422

**Published:** 2022-12-22

**Authors:** Wenzhang He, He Huang, Xiaoyi Chen, Jianqun Yu, Jing Liu, Xue Li, Hongkun Yin, Kai Zhang, Liqing Peng

**Affiliations:** ^1^Department of Radiology, West China Hospital, Sichuan University, Chengdu, China; ^2^Department of Cardiology, West China Hospital, Sichuan University, Chengdu, China; ^3^Infervision Medical Technology Co., Ltd., Beijing, China

**Keywords:** radiomics, aortic stenosis, left ventricular remodeling, cardiac magnetic resonance, TAVR, cine

## Abstract

**Objective:**

This study aimed to develop enhanced cine image-based radiomic models for non-invasive prediction of left ventricular adverse remodeling following transcatheter aortic valve replacement (TAVR) in symptomatic severe aortic stenosis.

**Methods:**

A total of 69 patients (male:female = 37:32, median age: 66 years, range: 47–83 years) were retrospectively recruited, and severe aortic stenosis was confirmed *via* transthoracic echocardiography detection. The enhanced cine images and clinical variables were collected, and three types of regions of interest (ROIs) containing the left ventricular (LV) myocardium from the short-axis view at the basal, middle, and apical LV levels were manually labeled, respectively. The radiomic features were extracted and further selected by using the least absolute shrinkage and selection operator (LASSO) regression analysis. Clinical variables were also selected through univariate regression analysis. The predictive models using logistic regression classifier were developed and validated through leave-one-out cross-validation. The model performance was evaluated with respect to discrimination, calibration, and clinical usefulness.

**Results:**

Five basal levels, seven middle levels, eight apical level radiomic features, and three clinical factors were finally selected for model development. The radiomic models using features from basal level (Rad I), middle level (Rad II), and apical level (Rad III) had achieved areas under the curve (AUCs) of 0.761, 0.909, and 0.913 in the training dataset and 0.718, 0.836, and 0.845 in the validation dataset, respectively. The performance of these radiomic models was improved after integrating clinical factors, with AUCs of the Combined I, Combined II, and Combined III models increasing to 0.906, 0.956, and 0.959 in the training dataset and 0.784, 0.873, and 0.891 in the validation dataset, respectively. All models showed good calibration, and the decision curve analysis indicated that the Combined III model had a higher net benefit than other models across the majority of threshold probabilities.

**Conclusion:**

Radiomic models and combined models at the mid and apical slices showed outstanding and comparable predictive effectiveness of adverse remodeling for patients with symptomatic severe aortic stenosis after TAVR, and both models were significantly better than the models of basal slice. The cardiac magnetic resonance radiomic analysis might serve as an effective tool for accurately predicting left ventricular adverse remodeling following TAVR in patients with symptomatic severe aortic stenosis.

## 1 Introduction

Aortic valve stenosis (AS) is one of the most prevalent heart valve diseases worldwide, with gradually increased morbidity with aging (1–3). Progressive pressure overload of the left ventricular to drive blood flow through the restricted left ventricular outflow tract, responsible for heart failure, is the threshold for myocardial decompensation, including myocardial cell damage and diffuse myocardial fibrosis, which exacerbates heart failure (3, 4). Then, the onset of symptoms is related to the rapidly increasing mortality of patients with a dismal prognosis (5). Valve replacement, providing a treatment method for advanced AS patients with complex physical environments, can prevent the further deterioration of heart function and improve myocardial remodeling even in the early stage (6–8). However, not all patients can benefit from valve replacement. Considering that advanced age, different underlying disease environments, long-term AS progression process, as well as diffuse myocardial fibrosis and impaired strain in patients with severe AS, the postoperative progress prediction based on a single image or clinical data remains uncertain (9–11).

The functional parameters of the left ventricle, especially left ventricular ejection fraction (LVEF), are important indicators for the evaluation of a patient’s preoperative status, perioperative risk, and postoperative recovery (12). In addition, myocardial strain, assessed by TTE, multiphase computed tomography, or cardiac magnetic resonance (CMR) cine sequence, can quantify percent change in myocardial length from relaxed to contractile state and reflect global and regional contractile function in longitudinal, circumferential, or radial directions (13, 14). CMR cine images have become the gold standard for left ventricular function and strain measurement, which is superior to transthoracic echocardiography (TTE) with restricted window width and more significant operator-to-operator differences. However, in fact, the biological information contained in the cine image is far more than the doctor can see with the naked eye.

Recently, R. Schofield et al. found that texture analysis based on cine images is of significance for the identification of the etiology of left ventricular hypertrophy (15), and Elham et al. made it clear that the radiomic signatures from cine images have the potential to detect myocardial ischemia, with the best area under the curve (AUC) of 0.93 (16). On account of computing power development and process standardization, the application potential of radiomics is being constantly mined in the cardiac field, including etiology determination, diagnosis confirmation, and prognosis prediction (17–21). For mechanical learning, a large amount of invisible biological information included in medical images was transformed into objective and quantitative digital information (22, 23). The research of Nam et al. has explored the excellent diagnostic efficacy of radiomics based on calcified plaques of the aortic valve for severe aortic stenosis with the highest AUC of 0.921 (20). Previous studies had also investigated the value of texture analysis in detecting left ventricular remodeling in cardiac computed tomography and CMR T1 mapping images (24, 25). However, whether the left ventricular remodeling following TAVR in patients with symptomatic severe AS could be predicted by radiomic analysis on enhanced cine images remained unclear.

In this study, our study aimed to develop a model for predicting left ventricular remodeling following TAVR in patients with symptomatic severe AS using the radiomic signatures from enhanced cine images.

## 2 Materials and methods

This study involving human participants was reviewed and approved by the Ethics Committee of West China Hospital. The need for informed patient consent was waived due to the retrospective nature of the analysis and the use of anonymized data.

### 2.1 Study population

We included 204 patients who underwent CMR before aortic valve replacement for symptomatic severe AS between October 2014 and June 2021. All patients performed heart-related symptoms such as dyspnea, angina, syncope, and dizziness (3). Additionally, all patients underwent TAVR. The author queried the patient’s echocardiographic recording from the Electronic Medical Records and searched for CMR images in the Picture Archiving and Communication Systems, and the time of CMR examination was within 7 days before TAVR, TTE within 3 days, and laboratory tests within 7 days. Excluding patients with TTE follow-up of less than 3 months and without adequate image quality, there were 156 patients.

**FIGURE 1:**
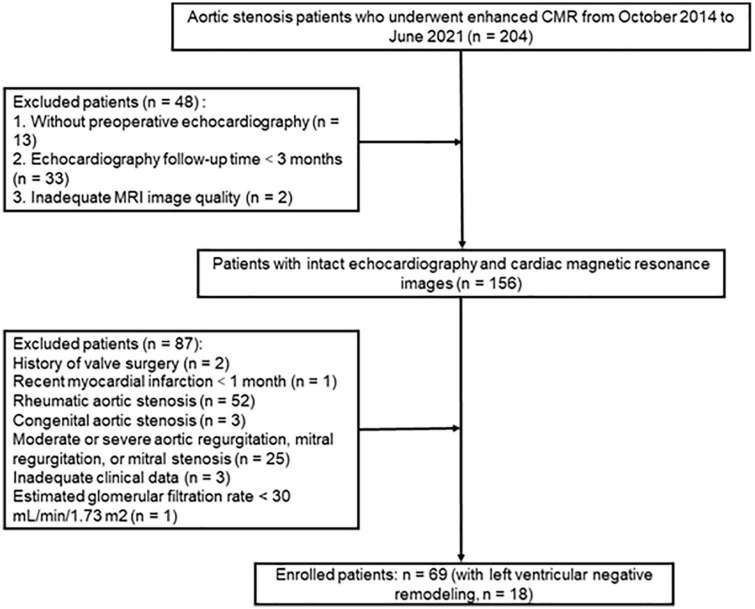
The patient selection workflow.

Each enrolled patient met the following exclusion criteria, and the exclusion criteria included: (1) history of valve surgery, (2) recent myocardial infarction <1 month, (3) congenital aortic stenosis, (4) moderate or severe aortic regurgitation, mitral regurgitation, or mitral stenosis, (5) inadequate clinical data, and (6) estimated glomerular filtration rate <30 ml/min/1.73 m^2^. Consequently, 69 patients, complying with our inclusion and exclusion criteria, consisted of study cohorts. All patients had follow-up TTE of more than 3 months, and the median follow-up duration was 12 months, ranging from 3 to 81 months. The patient selection workflow is shown in Figure 1.

### 2.2 Assessment of severe aortic valve stenosis

The severity of AS was assessed by echocardiography. Comprehensive transthoracic or transesophageal echocardiography examination was given to all patients. According to the recommendations of the current guidelines: mean gradient ≥40 mm Hg or peak aortic jet velocity ≥4.0 m/s and aortic valve area ≤1 cm^2^ (indexed aortic valve area by body surface area <0.6 cm^2^/m^2^ were diagnosed as severe aortic stenosis) (26).

### 2.3 TTE information

For patients with advanced aortic stenosis, accompanied by left ventricular decompensation, the patient manifests as a gradually enlarged left ventricle and a continuously reduced ejection fraction, and valve replacement is the only effective intervention (3, 26). After the TAVR, left ventricular remodeling is divided into three states, including adverse remodeling, relatively stable, and beneficial remodeling, and our study predicts further deterioration of left ventricular function during continuous follow-up, which is defined as adverse remodeling.

The measurements of the LVEF, left ventricular end-systolic diameter (LVESD), left ventricular end-diastolic diameter (LVEDD), left ventricular end-systolic dimension (LVESV), and left ventricular end-diastolic dimension (LVEDV) in preoperative and last follow-up TTE were collected. Patients with a relative increase of 15% in LVEDV or a relative decrease in LVEF of 10% were classified as subgroup 2 (with left ventricular adverse remodeling); otherwise, patients were categorized as subgroup 1 (with left ventricular non-adverse remodeling) (27).

### 2.4 Collection of clinical variables

A total of 14 clinical variables were collected from the electronic medical records, including two demographic characteristics (age and gender), one laboratory indicator (B-type natriuretic peptide [BNP]), three imaging features (myocardial hypertrophy, late gadolinium enhancement (LGE), and first-Pass perfusion), and eight cardiac function indicators measured in CMR (LVEF, LVEDV, LVESV, LVSV, RVEF, RVEDV, RVESV, and RVSV). The serum concentration of BNP was measured by electrochemical luminescence detection technology, and for patients with BNP serum concentration above the measurable extreme value of 35,000 pg/ml, we recorded it as 35,000 pg/ml.

Late gadolinium enhancement images were analyzed independently by a chest radiologist (with 5 years of experience with cardiac magnetic resonance) and then reviewed by another chest radiologist (with 12 years of experience with cardiac magnetic resonance). LGE is defined as a region with high signal within the myocardial region on LGE images and is clearly not due to image quality, artifacts, and partial volumetric effects.

### 2.5 Cardiovascular imaging – TTE and CMR

#### 2.5.1 Transthoracic echocardiography

Conventional two-dimensional TTE was performed using commercially available equipment. LV dimension and other echo parameters were obtained according to the guidelines of the American Society of Echocardiography (28). LVEDV and LVESV were measured in apical two- and four-chamber views, and LVEF was calculated using Simpson’s rule. The aortic valve area was calculated using the continuity equation, and the mean pressure gradient was calculated by averaging instantaneous gradients over the ejection period on the continuous wave Doppler recordings.

#### 2.5.2 CMR protocol – Imaging acquisition

Participants underwent CMR examination in a supine position. CMR images were acquired using a 3-T whole-body scanner (MAGNETOM Skyra; Siemens Healthcare, Erlangen, Germany) with an 18-channel phased-array body coil. Contrast medium (gadobenate dimeglumine; MultiHance; 0.5 mmol/ml; Bracco, Milan, Italy) was injected into the right antecubital vein with a power injector (Stellant; MEDRAD) at a flow rate of 3.0 ml/s, which was followed by injection of 20 ml saline. After first-perfusion images were collected, the enhanced cine sequence images were further scanned. With a standard ECG-triggering device, data were acquired during the end-inspiratory breath-hold period. A segmented breath-hold balanced steady-state free precession (bSSFP) sequence was used to obtain 8–14 continuous cine images from the base to the apex in the short-axis view and LV two- and four-chamber cine images in the long-axis view. The bSSFP parameters were as follows: TR/TE 3.3/1.22 ms; flip angle 41°; slice thickness 8 mm; field of view 360 mm × 320 mm; and matrix size 208 × 166. LGE images were obtained after contrast injection using a segmented phase-sensitive inversion recovery (PSIR) turbo FLASH sequence over a mean duration of 10–15 min. The PSIR sequence parameters were as follows: TR/TE 3.0/1.24 ms; flip angle 40°; slice thickness 8 mm; field of view 340 mm × 284 mm; and matrix size 256 × 184.

### 2.6 Image selection and segmentation

#### 2.6.1 Image selection

The cine sequence images of CMR are time series images at different levels, and the nature of non-spatial sequence images makes it difficult to segment wholeheartedly. Zahra et al. found that radiomic features from LV myocardium showed better repeatability in end-diastole rather than end-systole images (29). Therefore, we selected the enhanced cine images from the short-axis view at the basal, middle, and apical levels of the left ventricle in end-diastole. The definition of the basal, middle, and apical levels of the left ventricle at the short-axis view: (1) the first level of the appearance of left ventricular papillary muscle from the basal direction to the apical direction is defined as the basal level; (2) the second level below the basal level is defined as the middle level; and (3) the last level of the papillary muscle can be seen is defined as the apical level.

#### 2.6.2 Extraction and selection of radiomic features

**FIGURE 2:**
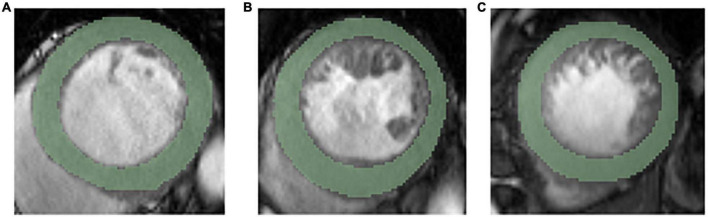
Representative ROIs segmentation in basal level **(A)**, middle level **(B)**, and apical level **(C)**.

The cine images in DICOM format from selected patients were segmented using “Radiomics” (Syngo. *via* Frontier, Vision 1.0.0, Siemens, Germany), a dedicated prototype software, and this program employs an embedded 3D-printing technique in a semiautomatic manner to label the preoperative soft tissue. The overall procedures of this analysis scheme were composed of two major steps: first, segmentation was conducted manually; and thereafter, texture features were calculated automatically. The manual segmentation was performed independently by a chest radiologist. The region of interest was depicted around the border of each level. After segmenting a three-dimensional volume of interest (3D-VOI), texture features were automatically calculated and extracted. Additionally, all cine images were segmented by two chest radiologists (with 6 and 12 years of experience with chest CT, respectively) working together. We completely outlined the left ventricular endocardial and epicardium at basal, middle, and apical levels and excluded the papillary muscles (Figure 2).

Before performing feature extraction, the 3D-VOIs were resampled to a pixel pitch of 1.0 mm in three anatomical directions to reduce the impact of pixel size and thickness. Through “bin the feature,” “radiomics” automatically transformed the grayscale of the image into discrete integer values, which were recognizable by a computer. In the original VOIs, different filters would be applied, such as the Laplacian of Gaussian filtering, wavelet filtering, non-linear intensity transformation, and others. We further excluded all shape- and size-related features as we only labeled isolated slices. Finally, a total of 1,302 radiomic features were extracted from each VOI, including 92 features extracted from the original image, and there were 93, 93, 93, 93, and 744 features extracted using the square, square root, logarithm, and exponential and wavelet filters, respectively.

The least absolute shrinkage and selection operator (LASSO) was used to reduce computation complexity and prevent overfitting. The key radiomic features most closely associated with the left ventricular adverse remodeling were selected with penalty parameter tuning conducted *via* a 10-fold cross-validation approach in the whole dataset.

### 2.7 Model development and evaluation

Two types of models were developed in this study, including three radiomics models based on the selected radiomic features and three combined models based on both selected radiomic features and clinical factors. The logistic regression classifier was applied for model construction, and the predictive models were developed and validated through leave-one-out cross-validation. Additionally, a final determined model could be generated by calculating the mean of the predictions by the models from each fold on a separate and independent validation dataset.

The performance of the predictive models was evaluated through the receiver operating characteristics (ROC) and quantified by the AUC. The sensitivity, specificity, positive predictive value (PPV), and negative predictive value (NPV) of each model were also calculated under the optimal threshold according to the maximum Youden index. Since the imbalance sample ratio in our study (18 adverse remodeling vs. 51 non-adverse remodeling), the accuracy and F1 score could lead to misleading results due to the lack of consideration for the ratio between positive and negative cases. Instead, as a robust index against class imbalance, the Matthews correlation coefficient (MCC) was used for binary classification evaluation in this study (30). The MCC ranged from −1 to 1, where −1 and 1 stood for complete misclassification and perfect classification, respectively, while 0 suggested that the model had no discriminatory ability.

### 2.8 Calibration and decision curve analysis

The consistency between the predicted adverse remodeling probability and the actual rate was assessed using the Hosmer–Lemeshow test, and the calibration curve was plotted using the 1,000 bootstrapping resampling method (31). The decision curve analysis (DCA) was applied to evaluate and compare the clinical usefulness of different models by estimating the net benefits across a reasonable range of threshold probabilities (32).

### 2.9 Statistical analysis

Statistical analysis was performed using the SPSS software (version 21.0). The differences between the continuous and dichotomous clinical variables were evaluated using the Mann–Whitney *U* test and the chi-squared test, separately (33). Delong’s test was applied for the comparison between the AUCs of two different models. The calibration curve and decision curve analysis were performed using the R language with the “rms” package and the “rmda” package, respectively. A two-sided *p*-value of <0.05 was considered statistically significant.

## 3 Results

### 3.1 Baseline clinical and TTE imaging parameters

We enrolled 69 patients (37 males) with symptomatic severe AS who underwent TAVR. The median and interquartile range for the age of all patients was 71.0 (66.0–75.0 [years]). The baseline characteristics of the study population are reported in Table 1. The median and interquartile ranges for LVEF and LVEDV were 62.0 (45.5–68.0) and 137.3 (102.0–171.0 [ml]), respectively. Additional TTE parameters and AV flow measurements are listed in Table 1.

**TABLE 1:** Clinical characteristics and echocardiographic parameters of the study cohort.

Age, years	71.0 (66.0–75.0)
Male gender	37 (53.6%)
Body mass index (Kg/m^2^)	23.2 (20.4–26.1)
Body surface area (m^2^)	1.7 (1.6–1.8)
Heart rate (bpm)	79.0 ± 2.7
Systolic blood pressure (mmHg)	129.7 ± 2.7
Diastolic blood pressure (mmHg)	72.3 ± 1.8
Tricuspid aortic valve	59 (85.5%)
**Past medical history**	
Atrial fibrillation	2 (2.9%)
Diabetes mellitus	8 (11.6%)
Hypertension	25 (36.2%)
Pulmonary hypertension	9 (13.0%)
Hyperlipidemia	3 (4.4%)
Chronic obstructive emphysema disease	8 (11.6%)
Smoker	18 (26.1%)
Drinker	12 (17.4%)
B-type natriuretic peptide (pg/ml)	3,445.8 (371.8–3,879.5)
Estimated glomerular filtration rate (ml/min/1.73 m^2^)	71.1 ± 2.1
Creatinine (umol/L)	98.1 (75.5–100.5)
**Diameters of aortic root and ascending aorta**	
Diameter of aortic annulus (mm)	32.5 ± 0.6
Diameter of sinus of Valsalva (mm)	22.1 ± 0.4
Diameter of ascending aorta (mm)	40.1 ± 0.7
**Baseline echocardiographic parameters**	
LVEF (%)	62.0 (45.5–68.0)
LVEDV (ml)	137.3 (102.0–171.0)
LVESV (ml)	50.0 (32.5–86.5)
LVEDD (mm)	50.0 (47.0–59.0)
LVESD (mm)	32.0 (28.0–42.0)
Interventricular septum (mm)	13.5 ± 0.3
Left ventricular posterior wall (mm)	11.5 ± 0.2
Left atrium size (mm)	40.1 ± 0.7
Em (m/s)	4.0 (3.0–5.1)
Am (m/s)	7.0 (5.9–9.3)
Emv/Em	17.0 (14.0–27.0)
**Parameters of grade of aortic stenosis**	
Max velocity of AV (m/s)	4.7 ± 0.3
Mean pressure gradient of AV (mmHg)	56.0 (45.0–68.0)
AVA (cm^2^)	0.7 ± 0.04

LVEF, left ventricular ejection fraction; LVEDV, left ventricular end-diastolic dimension; LVESV, left ventricular end-systolic dimension; LVEDD, left ventricular end-diastolic diameter; LVESD, left ventricular end-systolic diameter; AV, aortic valve; AVA, aortic valve area.

The median duration time from TAVR to last follow-up TTE was 12 months (interquartile range 6–13 months). Finally, according to the set criteria, 18 patients were defined as left ventricular adverse remodeling and 51 as non-adverse remodeling.

### 3.2 Comparison of CMR parameters between adverse and non-adverse subgroups

Compared with non-adverse remodeling subgroups, patients in the adverse remodeling subgroup showed a lower proportion of men, lower BNP levels, greater LVEF, smaller LVEDV, smaller LVESV, and smaller RVEDV, although there was no significant statistical difference in parameters other than BNP (*p* = 0.005). At the same time, the incidence of left ventricular hypertrophy was higher in the adverse remodeling subgroup. Other parameters did not show obvious distribution differences between the two subgroups. More detailed information is shown in Table 2.

**TABLE 2:** Comparison of clinical and CMR features between adverse and non-adverse subgroups.

	Non-adverse remodelling (*n* = 51)	Adverse remodelling (*n* = 18)	
Age, years	69.5 ± 10.1	69.3 ± 8.5	0.066
Male gender (%)	38 (74.5%)	9 (50%)	0.055
Heart rate (bpm)	76.0 (66.0–82.0)	72.5 (65.0–85.3)	0.806
Systolic blood pressure (mmHg)	126.9 ± 21.7	132.9 ± 13.5	0.177
Diastolic blood pressure (mmHg)	70.9 ± 14.1	75.4 ± 9.5	0.213
**NYHA**			
I	4 (7.8%)	3 (16.7%)	
II	16 (31.4%)	8 (44.4%)	0.162
III	21 (41.2%)	5 (27.8%)	
IV	9 (17.6%)	2 (11.1%)	
B-type natriuretic peptide (pg/ml)	2,586.0 (607.5–4,555.3)	576.0 (285.5–1,686.5)	0.005
LVEF	47.4 ± 19.5	56.1 ± 17.3	0.099
LVEDV (mL)	180.8 (126.1–227.4)	131.3 (107.2–206.8)	0.084
LVESV (mL)	86.3 (45.3–158.9)	57.0 (31.2–140.2)	0.159
LVSV (mL)	79.2 ± 23.4	77.5 ± 21.9	0.793
RVEF	54.3 (47.6–61.7)	55.4 (42.7–61.9)	0.929
RVEDV (mL)	95.5 (84.1–129.8)	85.6 (71.0–113.8)	0.060
RVESV (mL)	49.1 (29.4–66.2)	43.0 (29.1–60.9)	0.360
RVSV (mL)	51.5 ± 17.6	45.6 ± 15.5	0.209
**Myocardial deformation**			
0	27 (52.9%)	12 (66.7%)	
1	8 (15.7%)	3 (16.7%)	0.250
2	16 (31.4%)	3 (16.7%)	
Myocardial hypertrophy	20 (39.2%)	12 (66.7%)	0.045
LGE (+)	23 (43.1%)	8 (44.4%)	0.445
**LGE distribution**			
Basal	12 (23.5%)	7 (38.9%)	0.350
Mid	16 (31.4%)	8 (44.4%)	0.317
Apical	7 (13.7%)	3 (16.7%)	0.713
Septum	15 (29.4%)	8 (44.4%)	0.245
Free wall	16 (31.4%)	6 (33.3%)	0.878
Abnormal first-pass perfusion	3 (6%)	0 (0%)	0.704
Ascending aortic dilation	34 (66.7%)	8 (44.4%)	0.097
Calcification of ascending aortic wall	16 (31.4%)	8 (44.4%)	0.317

CMR, cardiac magnetic resonance; NYHA, New York Heart Association; LVEF, left ventricular ejection fraction; LVEDV, left ventricular end-diastolic dimension; LVESV, left ventricular end-systolic dimension; LVSV, left ventricular stroke volume; RVEF, right ventricular ejection fraction; RVEDV, right ventricular end-diastolic dimension; RVESV, right ventricular end-systolic dimension; RVSV, right ventricular stroke volume; LGE, late gadolinium enhancement.

### 3.3 Selection of radiomic features

**FIGURE 3:**
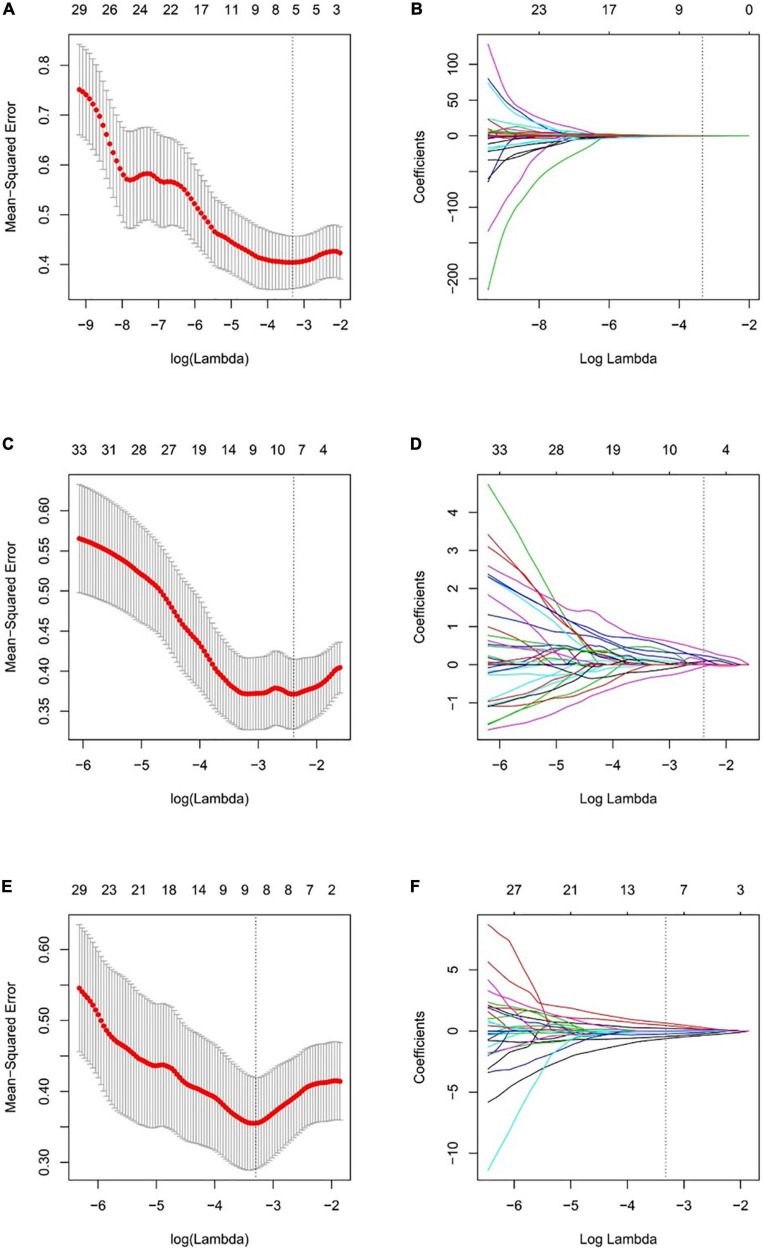
Selection of the radiomic features using LASSO regression. Selection of the optimal tuning parameter lambda through 10-fold cross-validation with the minimal mean-squared error criteria for the basal level **(A)**, middle level **(C)**, and apical level **(E)** radiomic features. **(B)** The coefficient profile plot of 5 in basal level **(B)**, 7 in middle level **(D)**, and 8 in apical slice **(F)** non-zero coefficients against the optimal log(lambda) sequence.

**FIGURE 4:**
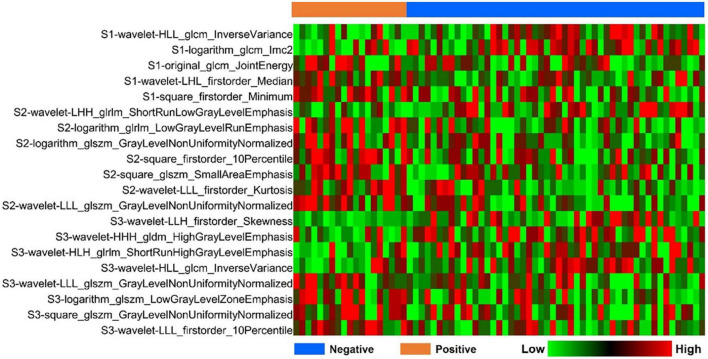
Heatmap analysis of the selected radiomic features. Each column corresponds to one patient, and each row represents one radiomic feature.

Based on the optimal log (lambda) sequence (−3.3155 for basal level features, −2.3908 for middle-level features, and −3.2976 for apical level features), 5 basal level features, 7 middle-level features, and 8 apical level features were selected for further analysis (Figure 3). The heat map of these selected key radiomic features was also plotted according to the normalized radiomic feature values (Figure 4).

### 3.4 Selection of clinical factors

To select the clinical variables mostly associated with pathological response, univariate regression analysis was performed (Table 3). Three clinical factors including BNP, LVEDV, and RVEDV showed a *p*-value < 0.05 and were selected for model development.

**TABLE 3:** Univariable analysis of clinical-radiological variables.

Variable	Univariate regression analysis		
	Odds ratio	95% CI	*P*-value
Age	0.9948	0.9429–1.0496	0.8486
Gender	0.6000	0.2071–0.7380	0.3465
BNP	0.9995	0.9991–0.9999	0.0203
LVEF	1.0309	0.9992–1.0636	0.0563
LVEDV	0.9785	0.9802–0.9992	0.0333
RVEDV	0.9785	0.9586–0.9988	0.0376

BNP, B-type natriuretic peptide; LVEF, left ventricular ejection fraction; LVEDV, left ventricular end-diastolic dimension; RVEDV, right ventricular end-diastolic dimension.

### 3.5 Comparison among three radiomic models

**FIGURE 5:**
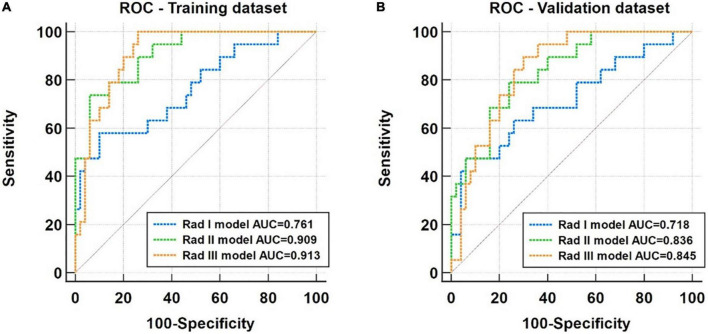
Receiver operating characteristic (ROC) analysis for performance comparison of the Rad I (basal level), Rad II (middle level), and Rad III (apical) models in the training dataset **(A)** and the validation dataset **(B)**.

The performance of the radiomic models based on selected features from the basal (Rad I model), middle (Rad II model), and apical (Rad III model) LV regions of interest (ROIs) is compared using ROC analysis in both training and validation datasets (Figure 5). The AUCs of the Rad I model, the Rad II model, and the Rad III model were 0.761 (95% CI, 0.643–0.856), 0.909 (95% CI, 0.816–0.965) and 0.913 (95% CI, 0.820–0.967) in the training dataset and were 0.718 (95% CI, 0.597–0.820), 0.836 (95% CI, 0.727–0.914), and 0.845 (95% CI, 0.738–0.921) in the validation dataset, respectively. Compared with the Rad I model, the AUCs of the Rad II model (*p* = 0.040) and Rad III model (*p* = 0.012) were significantly higher in the training dataset. A similar tendency was observed in the validation dataset; although not statistically significant, the AUCs of the Rad II model (*p* = 0.126) and the Rad III model (*p* = 0.103) were higher than that of the Rad I model.

### 3.6 Comparison between the radiomic model and combined model

**FIGURE 6:**
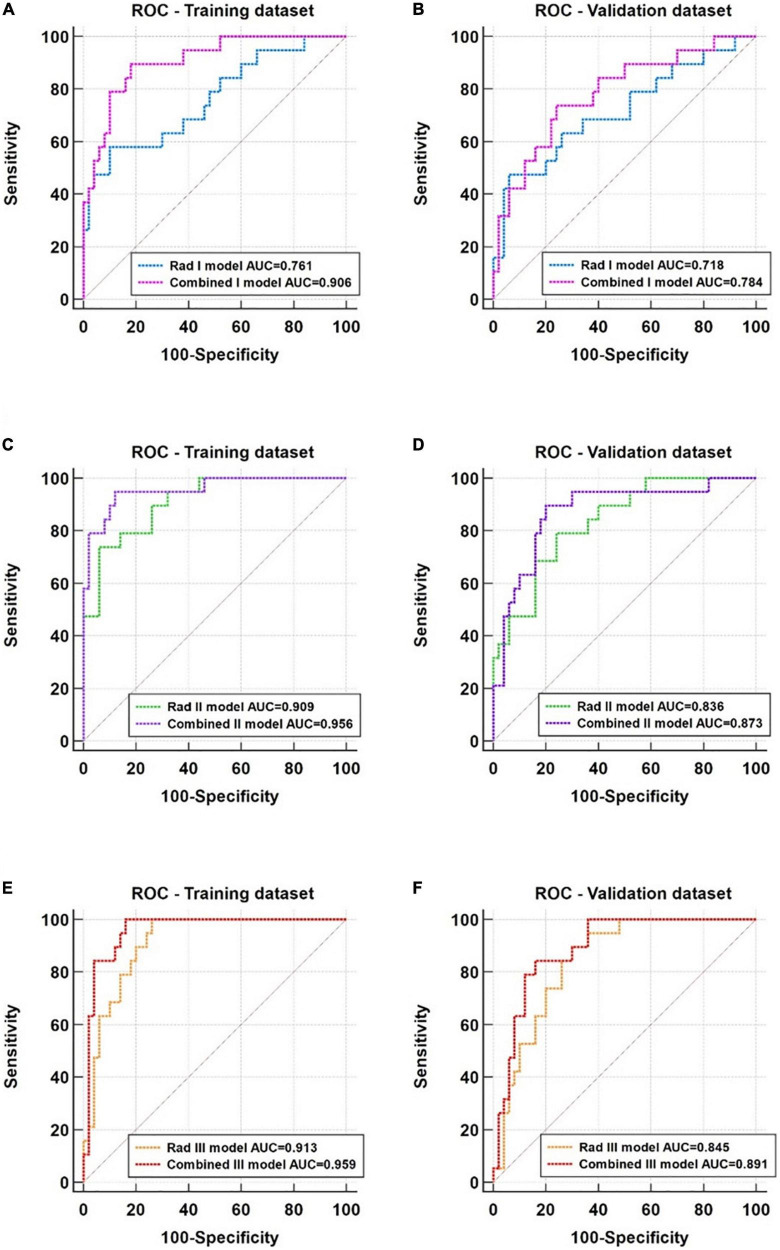
ROC analysis of the radiomic models and corresponding combined models. **(A,B)** Comparison of the Rad I (basal level) model and the Combined I model in the training and validation datasets. **(C,D)** Comparison of the Rad II (middle level) model and the Combined II model in the training and validation datasets. **(E,F)** Comparison of the Rad III (apical) model and the Combined III model in the training and validation datasets.

Based on the ROC analysis, the incorporation of clinical factors could improve the diagnostic capability of the radiomic models (Figure 6). Compared with the Rad I model, the AUCs of the Combined I model had increased to 0.906 (95% CI, 0.812–0.963) in the training dataset (*p* = 0.008) and 0.784 (95% CI, 0.669–0.874) in the validation dataset (*p* = 0.364). Compared with the Rad II model, the AUC of the Combined II model had increased to 0.956 (95% CI, 0.877–0.991) in the training dataset (*p* = 0.038) and 0.873 (95% CI, 0.770–0.941) in the validation dataset (*p* = 0.296). Compared with the Rad III model, the AUC of the Combined III model had increased to 0.959 (95% CI, 0.882–0.992) in the training dataset (*p* = 0.040) and 0.891 (95% CI, 0.792–0.953) in the validation dataset (*p* = 0.091). The Combined II model and Combined III model showed improved performance than the Combined I model; however, no significant differences in AUCs were found across the combined models in the training dataset (Combined I vs. Combined II, *p* = 0.125; Combined I vs. Combined III, *p* = 0.180; Combined II vs. Combined III, *p* = 0.922) and the validation dataset (Combined I vs. Combined II, *p* = 0.109; Combined I vs. Combined III, *p* = 0.090; Combined II vs. Combined III, *p* = 0.750). The detailed sensitivity, specificity, PPV, NPV, and MCC of these models are summarized in Table 4.

**TABLE 4:** Prediction effectiveness of radiomics and combined models in the training and validation cohorts.

Dataset	Model	AUC (95% CI)	SEN	SPE	PPV	NPV	MCC
Training cohort	Rad I	0.761 (0.643–0.856)	57.9%	90.0%	68.8%	84.9%	0.507
	Combined I	0.906 (0.812–0.963)	89.5%	82.0%	65.4%	95.3%	0.659
	Rad II	0.909 (0.816–0.965)	73.7%	94.0%	82.4%	90.4%	0.702
	Combined II	0.956 (0.877–0.991)	94.7%	88.0%	75.0%	97.8%	0.776
	Rad III	0.913 (0.820–0.967)	100.0%	74.0%	59.4%	100.0%	0.663
	Combined III	0.959 (0.882–0.992)	100.0%	84.0%	70.4%	100.0%	0.769
Validation cohort	Rad I	0.718 (0.597–0.820)	47.4%	94.0%	75.0%	82.5%	0.488
	Combined I	0.784 (0.669–0.914)	73.7%	76.0%	53.8%	88.4%	0.458
	Rad II	0.836 (0.727–0.914)	79.0%	76.0%	55.6%	90.5%	0.503
	Combined II	0.836 (0.727–0.914)	89.5%	80.0%	63.0%	95.2%	0.636
	Rad III	0.873 (0.770–0.921)	89.5%	70.0%	53.0%	94.6%	0.533
	Combined III	0.891 (0.792–0.953)	84.2%	84.0%	66.7%	93.3%	0.640

AUC, area under the curve; SEN, sensitivity; SPE, specificity; PPV, positive predictive value; NPV, negative predictive value; MCC, Matthews correlation coefficient.

### 3.7 Clinical utility evaluation in the validation dataset

**FIGURE 7:**
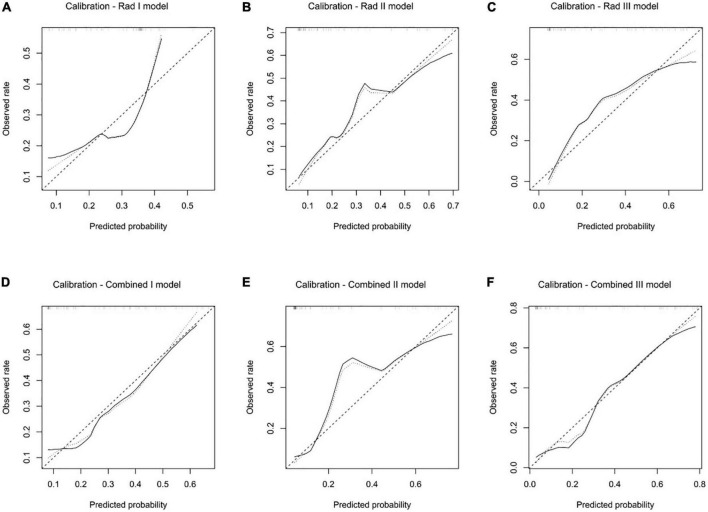
Calibration analysis of the Rad I model **(A)**, Rad II model **(B)**, Rad III model **(C)**, Combined I model **(D)**, Combined II model **(E)**, and Combined III model **(F)** in the validation dataset.

All the predictive models showed good consistency between the predicted left ventricular adverse remodeling probability and actual rate (Figure 7), and the non-significant statistic of the Hosmer–Lemeshow test suggested that there was no significant deviation from an ideal fitting of the Rad I model (*p* = 0.421), Rad II model (*p* = 0.328), Rad III model (*p* = 0.850), Combined I model (*p* = 0.903), Combined I model (*p* = 0.160), and Combined I model (*p* = 0.680) in the validation dataset.

**FIGURE 8:**
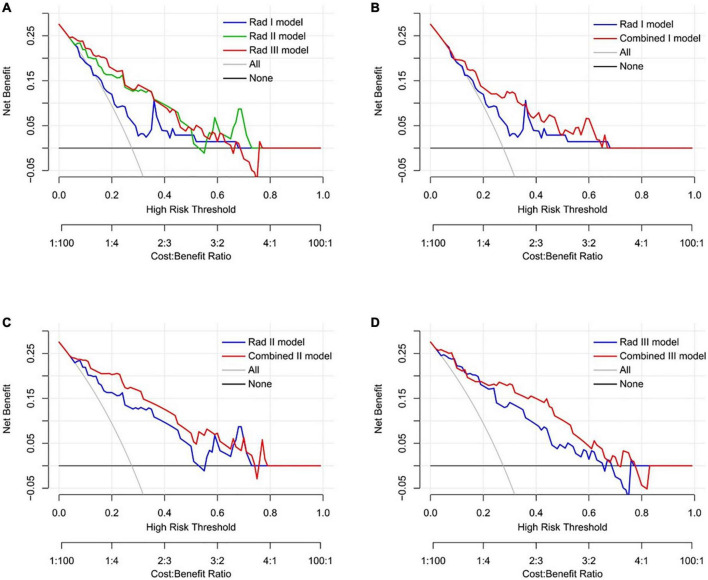
Decision curve analysis of the predictive models in the validation dataset. **(A)** Comparison across three radiomic models. **(B)** Comparison between the Rad I model and the Combined I model (basal level). **(C)** Comparison between the Rad II model and the Combined II model (middle level). **(D)** Comparison between the Rad III model and the Combined III model (apical level).

The decision curve analysis demonstrated that all the predictive models performed better than the treat-all and treat-none strategies (Figure 8). In addition, both the Rad II and Rad III models showed higher net benefit than the Stenosis I model across the majority range of threshold probabilities (Figure 8A). The net benefit of each combined model also increased compared with that of the corresponding Rad model, indicating that the incorporation of clinical factors could improve the clinical usefulness of the predictive model (Figures 8B–D).

## 4 Discussion

In the present study, ML-based texture analysis of enhanced cine images could accurately predict left ventricular adverse remodeling in patients with symptomatic severe AS after TAVR with AUCs ranging from 0.761 to 0.959 and 0.718 to 0.891 in training and validation cohorts, respectively. Of all the models, the apical level-based combined models exhibited the highest AUCs in both training and validation cohorts. Furthermore, the radiomic model based on apical slice exhibited the highest AUCs (0.913 in the training cohort and 0.893 in the validation cohort) and sensitivities (100.0% in the training cohort and 89.5% in the validation cohort) in training and validation cohorts of the three radiomic models in different levels. After adding three clinical features, three combined models in the basal, middle, and apical levels in the training set all showed better predictive performance than pure radiomic models in corresponding levels, which had higher AUCs (0.906 vs. 0.761, 0.965 vs. 0.909, 0.959 vs. 0.913), and performed similar trend in the validation set (0.784 vs. 0.718, 0.836 vs. 0.836, 0.891 vs. 0.873). These results confirmed that multivariate radiomic analysis is the potential to predict left ventricular adverse remodeling after TAVR in patients with symptomatic severe AS.

The excellent diagnostic effectiveness of our radiomic models may be the result of the excellent information-mining potential of radiomic analysis. Many past radiomic studies have found that radiomic signatures can catch differences in cell level, protein level, as well as gene level in tumors and non-tumor diseases (34–37). Both Roger et al. and Laurene et al. investigated tumor-infiltrating CD8 cells in different neoplasms through radiomics based on enhanced computed tomography and PRT-CT images (34, 35). Actually, in the process of left ventricular remodeling caused by aortic stenosis, changes in the molecular level, cell level, and interstitial level are the consequence of gene expression, which are further expressed as shape, size, as well as functional changes of the heart in the macro level (38). In clinical, due to various factors such as examination technology, iatrogenic trauma caused by examination, and patients’ willingness, it is difficult for doctors to distinguish the microscopic differences in patients with severe AS. Through imaging techniques such as CMR and TTE, we can quantify myocardial changes at the macroscopic level. Furthermore, due to the rapid development of CMR technology and its characteristic high tissue resolution, it has become a reality to non-invasively assess the microscopic changes in myocardial fibrosis (39, 40). LGE can characterize alternative fibrosis, which is the consequence of necrotic cardiomyocytes replaced by interstitial components (11). Additionally, T1 mapping as well as derived ECV technologies can quantify diffuse myocardial fibrosis degree, and both of them offer good consistency with myocardial biopsy (40). However, defects are also obvious. Myocardial interstitial fibrosis is not a simple increase in the number or volume of fiber structures but is accompanied by changes in fiber composition including the increase in the ratio of type 1 collagen fibers to type 3 collagen fibers and disorder of fiber arrangement structure (41). The models of myocardial fibrosis are not only diffuse fibrotic and alternative fibrotic models but also other models, such as alternative fibrosis and interstitial fibrosis mixing, are also being investigated (41, 42). The myocardial evaluation through CMR-related technologies has not yet reached this step. Differently, radiomic analysis based on cine images could detect myocardial infarction, with the best AUC of 0.93 and the best accuracy rate of 86% (16). The study by D. Alis et al. investigated ventricular tachyarrhythmia in hypertrophic cardiomyopathy by texture analysis of LGE images, with an AUC of 0.92 (43). The abovementioned study has verified that the texture characteristics based on CMR images can accurately capture patient-specific clinical information, which is of great value for patients’ personalized treatment planning. This may also be the reason why our radiomic models have excellent diagnostic efficiency. Unfortunately, since the nature of retrospective research, it is difficult for us to further verify our model at the gene level, cell level, or interstitial level, which is also a limitation of the research.

As a predictive factor for left ventricular remodeling after aortic valve replacement, global longitudinal strain (GLS) has a significant value, while LVEF has no clear predictive value (44–46). In AS, many scholars have found that GLS is mainly led by oriented longitudinally fiber shortening in the subendocardial layer where an early and selective alteration tends to happen (3). However, GLS measurement is influenced by complex factors, such as load, changes in cardiac morphology, and fluctuations in blood pressure as well as emotion (13).

In our study, three clinical factors including BNP, LVEDV, and RVEDV (all *p*-value < 0.05) were independent predictors of adverse remodeling of the left ventricle. Lower BNP levels, smaller LVEDV, and smaller RVEDV, associated with less myocardial injury, were found in the adverse remodeling subgroup. Giovanna et al. thought that BNP has a close relationship with AS severity, symptoms’ development, perioperative mortality, as well as ventricular remodeling (30). Previous findings that advanced myocardial disease with lower LVEF and lower GLS are associated with myocardial recovery demonstrate the potential benefit of TAVI (9, 47, 48). We conjectured that AS patients with serious myocardial damage who can withstand surgical trauma and perioperative period can acquire better benefits after the pathogenic factors are relieved. This phenomenon may be related to the characteristics of the patient’s own body.

Our study has a small study cohort, which may cause models to overfit in the training dataset. To alleviate this problem and completely use our data set, the leave-one-out cross-validation was applied to the modeling and verification process: one sample is taken as a validation dataset each time, the rest is used as a training dataset to train the model, and finally, the model was evaluated based on the validation results. SMOTE has not been used in our research to improve the distribution balance between groups. On the one hand, leave-one-out cross-validation was adopted in our models, and when newly amplified data through SMOTE was used as a validation set, the training set has the original data corresponding to the amplified data, which is equivalent to leaking part of the results to the artificial Intelligence in advance. On the other hand, in the research of D. Alis et al., there were similar problems of small sample size and uneven grouping, and their radiomic models with or without SMOTE showed excellent and comparable diagnostic effectiveness in ventricular tachyarrhythmia assessment with AUCs of 0.91 and 0.92, respectively (43). Therefore, we suggest that it is not a trivial step to solve the uneven distribution between groups in our study through SMOTE, which may have an impact on modeling. At the same time, in order to more accurately evaluate the predictive effectiveness of the model, we used MCC instead of F1 score or accuracy to evaluate the model to reduce the problem of inaccuracy caused by uneven distribution between groups (30).

The following limitations in our study should be acknowledged. First, selection bias may come from the research nature, a retrospective study from a single center. Second, since the acquisition method of CMR cine images is through slice-by-slice periodic acquisition, each image in any slice is the biological information of the myocardial tissue at the same level but at different points in the cardiac cycle, so our segmentation methods are to outline the left ventricle at a separate slice. Compared with the segmentation of the complete heart, our method may be relatively lacking in characteristic stability. Third, our research was only conducted on enhanced MRI images, and due to the influence of Gadolinium contrast agents, partial characteristics of the left ventricular myocardium may be buried. Fourth, as a result of a too small cohort, our research cohort was both used for training and validating models. The method of leave-one-out cross-validation was used to minimize the risk of overfitting. Modeling methods that have significant overfitting in the training set were excluded. Finally, we only included symptomatic severe AS patients with preoperative CMR, preoperative TTE and postoperative TTE follow-up of more than 3 months, and this may have resulted in selection bias because we excluded symptomatic severe AS patients with no preoperative CMR and TTE or without postoperative TTE follow-up.

## 5 Conclusion

The present study demonstrates that enhanced cine image-based texture analysis using logical regression algorithms is a promising tool for postoperative adverse remodeling prediction in patients with symptomatic severe AS. Logical regression-based quantitative analysis in apical slices with AUCs of 0.913 and 0.873 in training and validation groups was able to predict postoperative adverse remodeling in patients with symptomatic severe AS in the present study.

## Data availability statement

The original contributions presented in this study are included in the article/supplementary material, further inquiries can be directed to the corresponding author.

## Ethics statement

The studies involving human participants were reviewed and approved by the Ethics Committee of West China Hospital. The patients/participants provided their written informed consent to participate in this study.

## Author contributions

WH, HH, and LP: study design. WH and HH: data collection and data processing. All authors: manuscript writing, manuscript revision, final approval of the manuscript, and contributed to this study and approved the submitted version.
